# Highly Conductive Polyelectrolyte Membranes Poly(vinyl alcohol)/Poly(2-acrylamido-2-methyl propane sulfonic acid) (PVA/PAMPS) for Fuel Cell Application

**DOI:** 10.3390/polym13162638

**Published:** 2021-08-08

**Authors:** M. A. Abu-Saied, Emad Ali Soliman, Khamael M. Abualnaj, Eman El Desouky

**Affiliations:** 1 Polymeric Materials Research Department, Advanced Technology and New Materials Research Institute, City of Scientific Research and Technological Applications (SRTA-CITY), New Borg El-Arab City, Alexandria 21934, Egypt; emadasoliman@gmail.com; 2 Department of Chemistry, College of Science, Taif University, P.O. Box 11099, Taif 21944, Saudi Arabia; k.ala@tu.edu.sa; 3 Chemistry Department, Faculty of Science, Alexandria University, Alexandria 21568, Egypt; emanawad473@gmail.com

**Keywords:** PVA, PAMPS, PEM, DMFCs

## Abstract

In this study, chemically cross-linked PVA/PAMPS membranes have been prepared to be used in direct methanol fuel cells (DMFCs). The structural properties of the resultant membrane were characterized by use FTIR and SEM. Additionally, their thermal stability was assessed using TGA. Moreover, the mechanical properties and methanol and water uptake of membrane was studied. The obtained FTIR of PVA/PAMPS membranes revealed a noticeable increase in the intensity of adsorption peaks appearing at 1062 and 1220 cm^−1^, which correspond to sulfonic groups with the increasing proportion of PAMPS. The thermograms of these polyelectrolyte membranes showed that their thermal stability was lower than that of PVA membrane, and total weight loss gradually decreased with increasing the PAMPS. Additionally, the functional properties and efficiency of these polyelectrolyte membranes were significantly improved with increasing PAMPS proportion in these blends. The IEC of polymer blend membrane prepared using PVA/PAMPS ratio of 1:1 was 2.64 meq/g. The same membrane recorded also a methanol permeability coefficient of 2.5 × 10^−8^ cm^2^/s and thus, its efficiency factor was 4 × 10^5^ greater than that previously reported for the commercial polyelectrolyte membrane, Nafion^®^ (2.6 × 10^5^). No significant increase in this efficiency factor was observed with a further amount of PAMPS. These results proved that the PVA:PAMPS ratio of 1:1 represents the optimum mass ratio to develop the cost-effective and efficient PVA/PAMPS blend membranes for DMFCs applications.

## 1. Introduction

Hydrogen and DMFCs are common types of fuel cell that are considered a clean source of energy. The perfluorinated ionomer, DuPont’s Nafion^®^, that is made up of poly(tetrafluoroethylene) backbones with perfluoroetheral side chains ended use sulfonic acid [1] has been commonly applied in hydrogen as well as DMFC [2,3] owing to its special thermal, chemical, and mechanical stability, as well as its high proton conductivity. Notwithstanding, susceptibility to degrade at great temperatures, low ionic conductivity at low humidity, high methanol permeability, and deep- cost handicap its application in such technologies [4,5]. Therefore, many academic and industrial research groups have tried to develop polymer-based systems to produce PEMs as alternatives to replace Nafion. Nafion™ is a common material for PEM fuel cells, and it can be distinguished by its thickness and application. The membranes Nafion™ 115, Nafion™ 117, Nafion^TM^ 211 and Nafion^TM^ 212 are nonreinforced films with different thickness. The thickness chosen primary depends on its closeness to the prepared blended membranes [6]. Several polymers, either were natural or synthetic, have been functionalized with acidic, mainly sulphonic, groups via chemical modification or grafting, and blending of polymers or formation of composites of polymers has also been conducted with inorganic nanoparticles. Among the synthetic polymers that have drawn considerable attention in developing polyelectrolyte membranes, poly (vinyl alcohol) (PVA) is considered a cost-effective and polymer that is beneficial to the environment possessing strong film-forming characteristics, high hydrophilicity and chemical resistance [7,8]. In addition, methanol permeability of membranes PVA are smaller than that of Nafion^®^. Nevertheless, very poor proton conductivity of membrane PVA, determined as 2.45 × 10^−3^ S cm^−1^, was the main barrier to use in DMFCs application. The high density of hydrophilic functional –OH groups allows for chemical changes to the polymer chain by substitution, grafting, and crosslinking [9,10]. Therefore, these modification techniques, blending of PVA with acidic polymers and/or formation of its composites, have been reported to be feasible strategies to develop polyelectrolyte membranes. Blending of PVA with ionomers, such as perfluorosulfonic acid polymer (Nafion) [11], poly (styrene sulfonic acid) (PSS) [12], PAMPS [13,14], and chitosan [15], has been extensively reported to develop films with a high proton conductivity for fuel cell application. Among these ionomers, PAMPS has gained great attention because of its unique chemical structure accompanying its high water solubility and proton conductivity. Additionally, PAMPS has a lower cost compared with that of Nafion^®^117. However, PAMPS is unable to form self-supporting PEM because of its high solubility and thereby, it has to be blended with other supporting matrix. As a result of Qiao and his colleagues research, they have created a family of low-cost, easily manufactured PEMs based on chemically cross-linked PVA-PAMPS. It is worth noting that the membranes developed in this study have stronger proton conductivity and a lower methanol permeability coefficient than Nafion 117 [13,14]. However, the influence of polymer ratio on the blend membrane properties has not been investigated. Therefore, this study will be focused on this point to optimize the functional properties and performance of the glutaraldehyde-cross-linked blended proton-conducting membranes. The little amount of GA is entrapped in the host polymer of the blended membranes, PVA and PAMPS. GA is homogeneously diffused through membrane to improve mechanical characteristics and water resistance, and a chemical cross-linking reaction occurs between the aldehyde group (-CHO) of GA and the hydroxyl group (-OH) of PVA membrane to produce the acetyl ring. The sulfonic acid group (-SO_3_H) on PAMPS backbone along membranes by structural diffusion.

## 2. Materials and Methods

### 2.1. Materials

PVA (96.5% hydrolyzed, average Mw = 85,000–124,000) was acquired from Acros organics (Geel, Belgium). PAMPS (Mw ~ 2,000,000) and glutaraldehyde were acquired from Sigma-Aldrich (Taufkirchen; Germany) and Merck (USA), respectively. Sodium chloride was supplied by El Nasr Co. (Cairo; Egypt), sodium hydroxide from Fluka (Taufkirchen; Germany), ethanol from Doummar & Sons Co. (Taufkirchen; Germany), and methanol from El Salam Co. (Cairo; Egypt).

### 2.2. Preparation of PVA/PAMPS Blend-Based Membranes

In 10 mL deionized water, 1 gm of PVA was dissolved for 5 h at 800°C to produce a clear solution. Appropriate amounts of PAMPS were dissolve in 10 mL deionized water at ambient temperature. The particular molar ratio of solid polymer membrane PVA–PAMPS was 1:3, 1:2, 1:1.5, 1:1, and 1: 0.5, respectively. Cross-linking was used to optimize the resulting polymer blend membranes for fuel cell applications. For this, 0.1 mL GA (25 wt.% in water) were incorporated into polymers blend solutions to create a chemically constrained network through the cross-linking of PVA chains. Finally, the obtaining homogeneous, transparent and viscous film-forming solution was put into plastic Petri dishes after removal of the air under vacuum, as well as the water evaporating at room temperature when the membrane appeared to be dry, the film was pulled away [16,17].

### 2.3. Characterization of PVA/PAMPS Blend-Based Membrane

#### 2.3.1. FTIR

Before spectroscopic measurement, all polymeric membrane was kept in desiccators including at least 48 h of silica gel at room temperature to eliminate moisture content. Shimadzu 8400S spectrometer (Shimadzu, Kyoto, Japan )fitted with a Golden Gate diamond horizontal attenuated total reflectance (ATR) system was used to investigate the non-cross-linked PVA, GA-crosslinked PVA, and PVA/PAMPS blend membrane specimens. On the ATR crystal, a membrane specimen (10 mm 10 mm) was put to fill the crystal sur-face region. A screw gently squeezed the speci-men to promote contact with crystal. The spectra was taken with a range of 4000–400 cm^−1^. The ATR crystal had a 45° angle of incidence. As a backdrop, an empty crystal was employed. The Fourier-deconvoluted FTIR spectra had a resolution enhancement factor of 1.5 and a bandwidth of 15 cm^−1^ [18,19].

#### 2.3.2. SEM

A Joel 6360LA scanning electron microscope (JEOL Ltd., Tokyo, Japan) were used to analyze the surface morphology of polymeric membrane at a 10 kV acceleration voltage. Membrane specimens were double-sided taped on stainless steel stubs, and a 10–20 nm thick layer of gold were sputtered on the samples with a JFC-1100E sputter (JOEL Ltd., To-kyo, Japan) [18].

#### 2.3.3. Thermogravimetric Analysis

Thermogravimetric study of the resultant polymeric blend films was occurred using Shimadzu TGA-50 (Shimadzu, Japan) under nitrogen. The film samples of approximately 5 mg are put within aluminum pans, and heated from the ambient temperature to 800 ºC at a rate of 10 ºC min^−1^ [20,21].

#### 2.3.4. Tensile Testing

The tensile strength (TS) and elongation at break (E) of the membrane were determined using a universal testing machine (model AG-I, Shimadzu, Kyoto, Japan). The initial grips separation was set to 10 cm, and the cross-head speed at 1 mm/min. Prior to tensile testing, film specimens was conditioned for two days in an environmental room at 22 2 °C and 70% RH using ASTM Standard Method D 882-91(ASTM 2011). For each type of generated polymeric membrane, the TS, E values were calculated for each made cast membranes as duplicated experimental units. The mean of five experimental units was used to calculate each TS, E replicate value (specimens) [17,22].

#### 2.3.5. Water Contact Angle Measurement

The inner angle produced by the membrane’s surface and the tangent to the surface of liquid droplets indicating hydrophobicity or wettability on the membrane’s surface is referred to as the contact angle. The contact angles with a water droplet were measured by use a goniometer (Ramé - Hart, model 500-F1, France). A 4 L droplet of de-ionized water was placed on the suction with an automatic piston syringe [23].

#### 2.3.6. Ion Exchange Capacity

The quantity of sulfonic groups in the membrane determines the ion exchange capacity (IEC). Membrane samples were submerged in a NaCl solution (2M) for 24 h to be evaluated. The protons released from these materials were determined using acid-base titration with 0.01N sodium hydroxide and phenolphthalein as an indicator. The IEC of polyelectrolyte was calculated using equation below [23,24].

$$\mathrm{IEC}\;\left(\mathrm{meq}/\;g\right)=\frac{N\;x\;V}{W\;}$$

where V and N are the NaOH volume titer and solution’s normality, respectively. W is the weight of the dried membranes sample that were placed in an oven at 50°C for 12 h before being weighed.

#### 2.3.7. Methanol and Water Uptake

For 24 h, the membrane specimen was immersed in deionized water at ambient temperature before being removed from the water. The following equation was used to compute water uptake:

$$W\;\left(\%\right)=\frac{W_{\mathrm{wet}\;}-W_{\mathrm{dry}\;}}{\;W_{\mathrm{dry}}}$$

The dry weight of the membrane (*W*_dry_) was calculated by drying the membrane sample for 12 h at 50 °C before weighing it. The weight of the membrane in the wet state (*W*_wet_) was determined by (i) immersing the identical membrane samples in distilled water for 24 h and (ii) promptly wiping off the surface moisture with filter paper before weighing the sample was obtained by immersing an identical membrane samples in distilled water for 24 h and then wiping off the surface moisture with filter paper before weighing it [25].

For testing methanol uptake, the standard approach of replacing methanol with water was used [26,27].

#### 2.3.8. Methanol Permeability

Methanol permeability was measured by the tested film separated two-glass compartments in a glass diffusion cell. Compartment A with volume of 125 mL was occupied with methanol (15 wt.%), and compartment B with volume of 120 mL was full with deionized water. The permeation process was undertaken under stirring with taking liquid sample of 1 mL from permeate at intervals of 10 min to determine the amount of permeated methanol using Anton paar instrument. Methanol permeability (P) was calculated from the obtained equation:

$$P=\frac{K\times V\times L}{A\times C_{A}}$$

where *K* = the slope of permeation curve, *V =* the initial volume of deionized water, *L* = the thickness of membrane, *C_A_* = the initial concentration of methanol, and *A* = the area of permeation.

#### 2.3.9. Membrane Efficiency

The effectiveness of polyelectrolyte membranes to be employed in DMFCs can be estimated using the following equation, which expresses their ionic conductivity and methanol permeability [28].

$$ɸ=\frac{\sigma }{P}$$

where is a ɸ that indicates membrane efficiency as a ratio of ionic conductivity (σ) to methanol permeability. However, in this study, IEC was proposed to be utilized in place of ionic conductivity in this equation, as shown below [20,29]:

$$ɸ=\frac{IEC}{P}$$

## 3. Results and Discussion

### 3.1. ATR-FTIR Spectra

**Figure 1:**
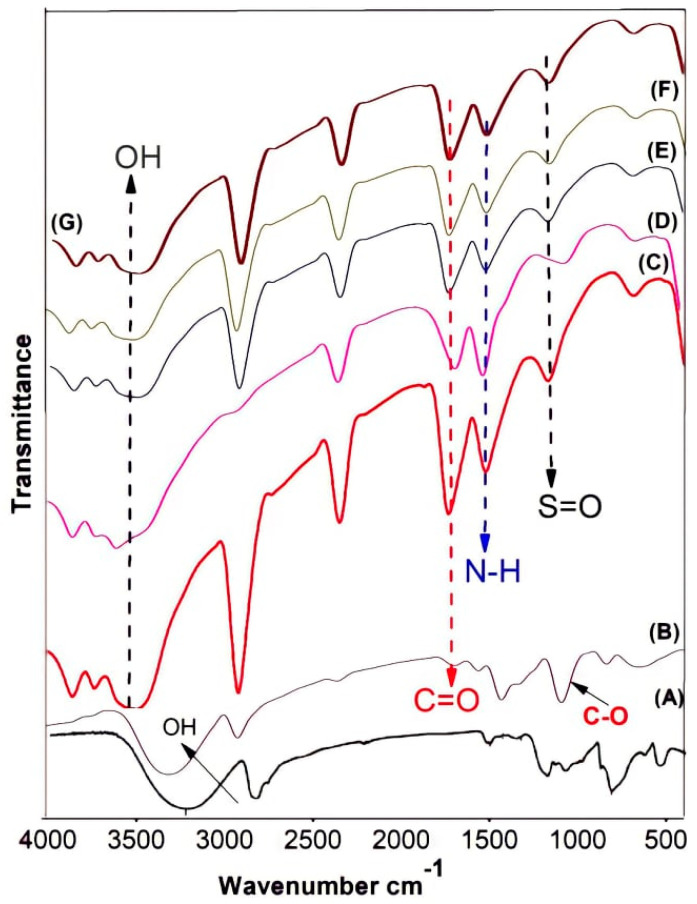
ATR-FTIR spectra of non-cross-linked PVA (**A**), GA-cross-linked PVA (**B**), and GA-cross-linked PVA/PAMPS blend-based membranes prepared using polymer ratio of 1:0.5 (**C**), 1:1 (**D**), 1:1.5 (**E**), 1:2 (**F**), or 1:3 (**G**).

ATR-FTIR spectra of pristine PVA, GA-cross-linked PVA, and PVA/PAMPS blend-based membrane was obtainable in Figure 1. The spectrum of non-crosslinked PVA membrane shows a characteristic peak at 2926 cm^−1^ due to asymmetrical stretching of C–H bonds, as well as a strong signal at 3276 cm^-1^ due to O–H stretching vibrations. Meanwhile, the spectrum of cross-linked PVA reveals another absorption characteristic peak at 1701 cm^−1^ due to stretching C=O belonging to aldehyde groups of glutaraldehyde molecules, which attached to hydroxyl groups of PVA with one aldehyde group while leaving the other one free due to no other close PVA molecules for achieving the cross-linking process. Moreover, this cross-linking was accompanied by the appearance of an adsorption peak due to C–O stretching at 1105 cm^−1^. By comparing spectra of polymer blend membrane with that for GA-cross-linked PVA membrane, new absorption bands are noticed at 1021 and 1112 cm^−1^ due to the symmetric and asymmetric O=S=O stretching vibrations of –SO_3_H groups belonging to PAMPS, respectively. Such adsorption peaks could overlap those ascribed to n(C-O) formed in an ester mode. Moreover, other sharp adsorption peaks appear at 1650 cm^−1^ and 1545 cm^−1^, which are due to vibration of C=O and N–H groups in PAMPS, respectively. However, the absorption peaks at 3306 cm^−1^ due to stretching of O–H appear in all spectra, where they are belonging to PVA. The intensity of this peak is low in case of the pristine PVA or PVA/PAMPS blend-based membranes due to the chemical cross-linking. On the other side, the adsorption band assigned to N–H stretching has not clearly appeared in the blend membrane spectra because it is interfering with those corresponding to O–H groups [27].

### 3.2. Morphological and Cross-Sectional Features

**Figure 2:**
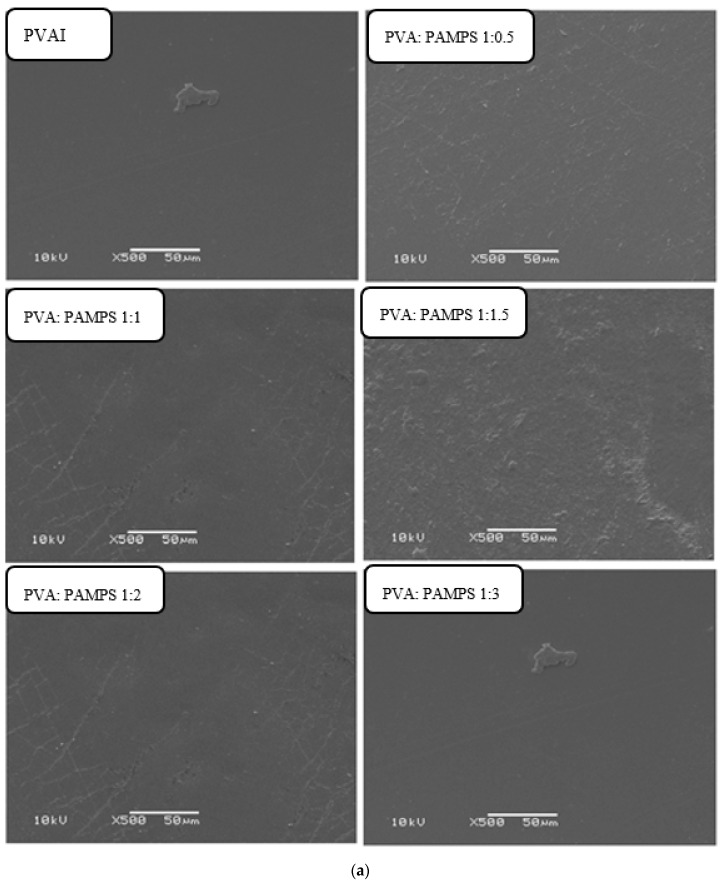

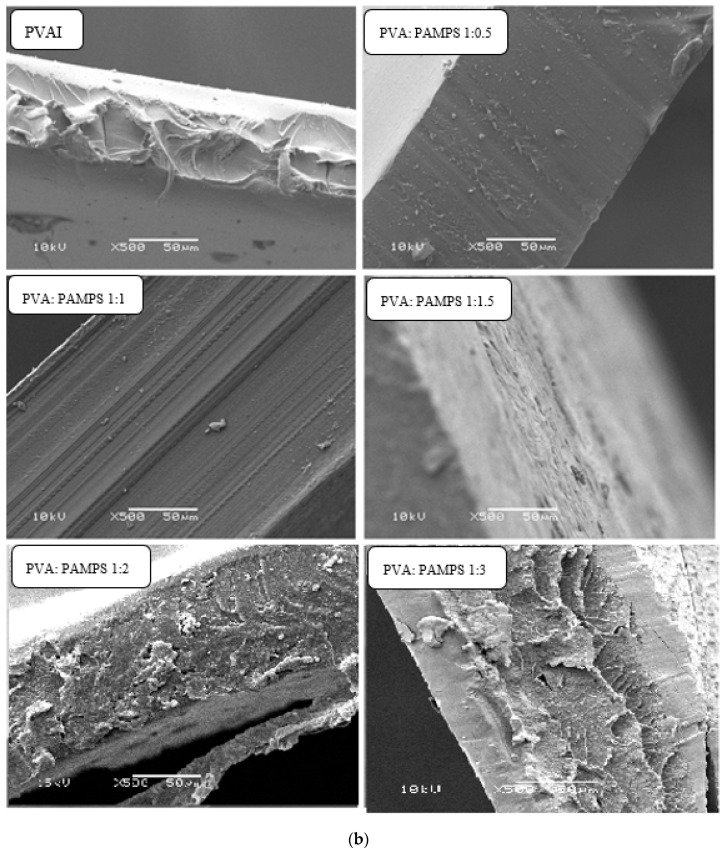
(**a**): SEM micrographs of surface of pristine PVA and PVA/PAMPS blend-based membranes at magnification power of 500×. (**b**): SEM micrographs of cross-section of pristine PVA and PVA/PAMPS blend-based membranes at magnification power of 500×.

SEM micrographs of the surface and cross-section of pristine PVA and GA-cross-linked PVA/PAMPS-based membranes prepared with different blend ratios were presented in Figure 2a,b, respectively. The surface micrographs revealed that the pristine PVA-based membrane has a smooth and even surface. Apparently, blending of PAMPS with PVA in the blend membranes led to the appearance of a slightly more topographic terrain, making the membranes surface uneven. Therefore, a homogeneous distribution for these morphological features with no phase separation in these blend membrane matrices was observed, indicating the miscibility of these two main polymers. Therefore, it was indicated that the PAMPS blended well with PVA at the applied different ratios. The cross-sectional SEM micrographs of pristine PVA membrane display a highly tight and dense structure. However, the inclusion of PAMPS into blend membrane matrices led to the formation of loose structure with wrinkled fractures. This looseness increased with increasing the proportion of PAMPS in the blend membrane. These results can be attributed to staking of extended PVA layers in pristine PVA membrane matrix. Nevertheless, entanglement of PVA and PAMPS chains in the blend membrane matrices can hinder the intermolecular hydrogen bonding and crystallinity of PVA molecules and alignment of PVA/PAMPS layers by the action of the dimethyl and sulphomethyl groups of anionic polyelectrolyte, PAMPS, which sterically hinder the potential molecular interactions between the PVA chains or layers [30].

### 3.3. Thermal Stability

**Figure 3:**
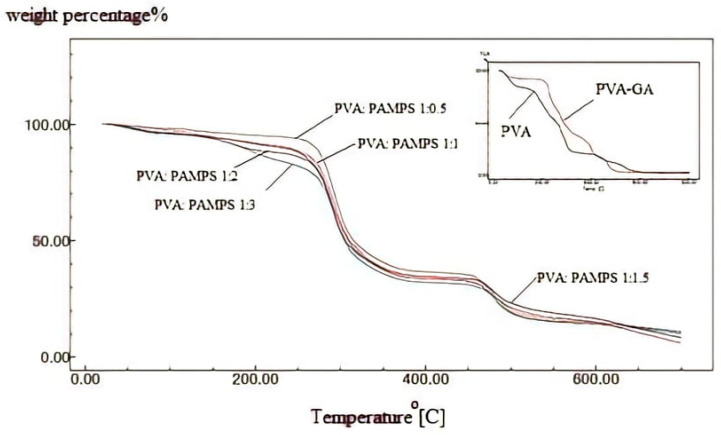
TGA Thermograms of non-cross-linked PVA, GA-cross-linked PVA, and PVA/PAMPS blend-based membranes.

The thermal stability play a crucial role in determining the operating life of the polyelectrolyte membrane in fuel cell application. TGA thermograms of non-cross-linked PVA and GA-cross-linked PVA and PVA/PAMPS blend membranes prepared by using different ratios of PVA and PAMPS are shown in Figure 3. The variation in the residual weight percentages with temperature described in the thermograms is tabulated in Table 1. The thermogram of non-cross-linked PVA revealed three main thermal degradation stages. The first stage is distinguished by the peak at around 100 °C with weight loss of 37% of the initial weight. This is ascribed to loss of water molecules trapped or adsorbed in the hydrophilic PVA matrix. The second step of weight loss, which occurred at temperatures ranging from 230 to 340 °C, is caused by the thermal degradation of PVA chains with splitting –OH groups. The third degradation stage observed at 350–600 °C apportioned to the decomposition of the PVA backbone. Moreover, TGA thermogram of glutaraldehyde-cross-linked PVA exhibited three degradation stages in three different temperatures ranges. The first weight loss of about 10% was in the range 50–180 °C is ascribed to the vaporization of the trapped water or release of the absorbed moisture. The second stage of weight loss, which was about 50%, occurred between the temperatures 250 and 450 °C and can be assigned to the dehydration and splitting of PVA chains, i.e., degradation of the polyene residues to yield hydrocarbons (volatile products including acetaldehyde and croton aldehyde) and carbon. The third stage of weight loss, which was about 18%, happened from temperature 400 to 580 °C as a result of the destruction of cross-linking bridges. The residual weights in such glutaraldehyde-cross-linked PVA membrane were higher than those of non-cross-linked PVA membrane. These findings indicate that the cross-linking lowers the thermal degradation rate of PVA membrane. TGA curves of glutaraldehyde-cross-linked PVA/PAMPS blend-based membranes, on the other hand, revealed a nearly three-stage decomposition process, with the first stage, occurring at 50–200 °C with approximately 17–25 percent loss of initial weight, thought to be the evaporation of residual water present in the polymer matrix. The greatest weight loss, approximately 40–45 percent, happened in the second stage of decomposition, which began at 200 °C and ended at roughly 480 °C. This is due to the decomposition of sulfonic acid groups (SO_2_ and SO_3_) and the cleavage of the PVA chain’s backbone. This stage was followed by a further 15–30% loss of membrane weight between 450 and 650 °C due to breaking of cross-linking and decomposition of the main polymeric chains. Generally, it was observed the thermal degradation rate for these blend membranes was higher than that of GA-cross-linked PVA membrane. Furthermore, the residual weight percentages at different thermal degradation temperatures were decreased with increasing the mass of PAMPS. On the contrary, the previous study of Qiao and his colleagues reported that the reported that combination of PAMPS into PVA matrix enhanced the thermal stability of the resulting blend membranes [13]. These findings can be explained on the basis that no association occurred between the polymer components, and thus the higher degradation of PAMPS compared with the cross-linked PVA chains caused an increase of the thermal degradation polymer blends with increasing the mass content of PAMPS.

### 3.4. Mechanical Properties

The mechanical characteristics are considered one of the key factors in deciding the potential feasibility of PEM in DMFCs. The mechanical properties of chemically cross-linked PVA and PVA/PAMPS mix mem-branes for tensile strength and % elongation at break were examined, and the findings are shown in Table 2. TS of glutaraldehyde-cross-linked PVA membrane of 20 Mpa was greater than that for all glutaraldehyde-cross-linked PVA/PAMPS blend based membranes. Furthermore, these tensile properties gradually decreased with increasing the mass ratio of the PAMPS. Still, the blend based membrane specimens prepared at PVA:PAMPS ratio of 1:0.5 are ductile this can fulfill the membrane electrode assembly (MEA) requirement) [27].

### 3.5. Contact Angle

The hydrophilicity of a polyelectrolyte membrane surface is an indicator for water swell ability or uptake that is related to water stability, IEC, and proton conductivity of this membrane. Thereby, this property was assessed by measuring the water contact angle. Water contact angle of chemically cross-linked PVA and blended PVA/PAMPS membranes is displayed in Table 2. These results show that the contact angle of glutaraldehyde-cross-linked PVA membrane was 34.05°. Moreover, blending anionic water-soluble polymer, PAMPS, with PVA lowered the contact angle of the resulting membranes. This decline in contact angle was raised when the quantity of PAMPS was increased to reach up to 18.31° for membranes prepared at PVA: PAMPS ratio of 1:3. This conclusion can be illuminated by the hydrophilicity of PAMPs, which is attributable to the presence of sulfonic acid groups in their chemical structure [16].

### 3.6. Functional Properties

#### 3.6.1. Water and Methanol Uptake

The proton conductivity of PEM is strongly dependent on its water content, and hydration of the PEM is a crucial key to improve proton conductivity and hence the effectiveness of the fuel cell. Table 2 shows the water uptake of GA-cross-linked PVA and PVA/PAMPS blend based membranes [20].

Methanol moves from the anode to the cathode with the help of water during operation of DMFC. Therefore, methanol sorption and uptake of these PEMs must be low in order to prevent fuel crossover. Hence, methanol uptake for GA-cross-linked PVA and PVA/PAMPS prepared membrane was determined, and the results are scheduled in Table 2. These data indicate that glutaraldehyde-cross-linked PVA membrane had methanol uptake of about 17.5%. Furthermore, blending membrane PAMPS with PVA led to lowering of the methanol uptake of the resultant polymeric membranes. Uptake of methanol was gradually decreased with rising the quantity of PAMPS in the blend to reach up to 9.5% for blended membrane prepared at PVA/PAMPS ratio of 1:3 [31]. The high hydrophilicity of the chemically cross-linked PVA and PVA/PAMPS mix mem-branes can be attributable to these results. Whereas the amount of PAMPS in the mem-brane matrix increased, the hydrophilicity of this blended membrane increased due to the hydrophilic sulfonic groups and, as a result, methanol sorption reduced.

#### 3.6.2. Ion Exchange Capacity (IEC)

IEC is a symptom for the presence of exchangeable protons or sulfonic groups on polymer matrix, which are relevant to proton conduction, and is thus an indirect and reliable estimate for proton conductivity. The IEC of chemically cross-linked PVA and PVA/PAMPS prepared membranes was estimated at room temperature, and the IEC values are presented in Table 2. The results show that GA-cross-linked PVA membrane had the lowest IEC (0.08 meq/g). Some of the prepared films had IEC values higher than that for modified (PVC-PAMPS 1:3) membrane (1.25 meq/g) and Nafion-117 (0.91 meq/g). Moreover, IEC values were significantly increased with the assigned up of PAMPS content (i.e., the sulfonic groups) in the membrane. The maximum IEC value (3.93 meq/g) was recorded for prepared membrane with PVA: PAMPS ratio of 1:3 [32].

#### 3.6.3. Methanol Permeability

**Figure 4:**
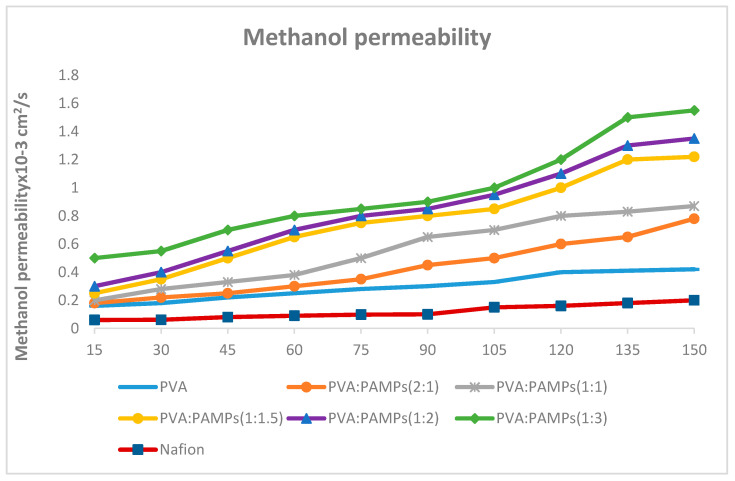
Amount of permeated methanol as a function of time for PVA and PVA/PAMPS blended membrane.

Permeation of methanol across PEM during DMFCs operation causes waste of the fuel and harming of the catalyst, thus decreases the proficiency of fuel cells. As a consequence, the methanol permeability of GA-cross-linked PVA and PVA-PAMPS polymer blend was measured in a diffusion cell with the membrane clamped between two reservoirs of aqueous methanol solution and distilled water. The quantity of permeated methanol was plotted versus the time, and the results are presented in Figure 4. The methanol permeability coefficient can be determined using the acquired line’s slope and the values in Table 2. These findings show that chemically cross-linked PVA membrane had the methanol permeability coefficient of 4.8 × 10^−7^ cm^2^/s, which was lower than that of Nafion^®^117 (3.39 × 10^−6^cm^2^/s). In addition, the chemically cross-linked PVA/PAMPS blended membranes revealed lower methanol permeability comparing with that of GA-cross-linked PVA membranes. Contrary to the methanol uptake results, the methanol permeability coefficient was noticeably enlarged with increasing the content of PAMPS to reach up to 3.2 × 10^−8^ cm^2^/s for prepared membrane using PVA:PAMPS ratio of 1:3. These finding can be explained as the content and state of water (freezing and nonfreezing) in the prepared membrane matrix from one side and the type of dominant attractions between the methanol molecules in the aqueous solution, either hydrophobic or hydrophilic, governing its association dynamics, and thereby, its permeation, on the other side [13,33]. These effects can be highly entangled and complicated during the DMFCs operation due to elevating the temperature.

#### 3.6.4. Membrane Efficiency

Efficient PEM for DMFCs operation should simultaneously possess high proton conductivity with a poor permeability to methanol That is, the proton conductivity to methanol permeability ratio is more essential than methanol permeability to some extent. The efficiency of the resulting membranes in this study was assessed by measuring the efficiency factor (ɸ), as a ratio of IEC to methanol permeability. The obtained results are presented in Table 3. These data indicate that the ɸ value of all chemically cross-linked PVA/PAMPS blended membranes was greater than that of chemically cross-linked PVA membrane (1.10 × 10^5^). Furthermore, the ɸ value of these blend membranes increased with increase as the quantity of PAMPS in the blend increases. The top value (4.70 × 10^5^) was gained for blend membrane prepared at PVA:PAMPS ratio of 1:3. In the same context, it is worthy to mention that the ɸ value of all blended membranes prepared using equal proportion of main polymeric components, PVA and PAMPS, or by using higher percentages of the latter was higher than that of Nafion^®^ 117 (2.60 × 10^5^). However, the most efficient polymer blend membrane was indicated to be that fabricated using PVA:PAMPS ratio of 1:1, and any excess in PAMPS over this percentage did not cause any significant enlargement in the membrane efficiency, and thus can be considered unjustified cost [34].

## 4. Conclusions

This study concentrates on the preparation of glutaraldehyde-cross-linked PVA/PAMPS prepared membranes using varying ratios of PVA and PAMPS to optimize the membrane properties and performance in DMFCs operation.

The compositional and structural properties of chemically cross-linked PVA/PAMPS blend polyelectrolyte were study by use FTIR and SEM techniques. FTIR confirm the attainment of cross-linking of PVA by use GA and also show intermolecular connection between the polymer constituents. Moreover, SEM micrographs reveal that there was no any interphase incompatibility or phase separation in the matrices of all polymer blend proton-conducting membranes prepared using the tested varying polymer ratios.

The incorporation of PAMPS into the chemically cross-linked PVA matrix enhanced the IEC, WU, and thermal stability of the resulting polyelectrolyte membrane and simultaneously lowered their methanol uptake and permeability. However, this blending process led to decreasing the mechanical strength.

On the other side, the findings of this study show that an increase in the PAMPS content led to a gradual increase in the IEC, WU, and methanol permeability. However, the thermal stability, mechanical strength, and MU were gradually decreased. Such results are supportive of enhancing the efficiency of the obtained polymer blend proton conducting membranes. To optimize the performance and efficiency of these polymer blend membranes in DMFCs operation, PVA: PAMPS ratio of 1:1 was proposed to be the ideal. The resultant membrane at this ratio showed IEC of 2.64 meq/g and methanol permeability measurement of 2.5 × 10^−8^ cm^2^ s^−1^, and thus the efficiency factor was 4.00 × 10^5^, which is greater than that of commercially Nafion^®^117. Moreover, the tensile strength of this membrane was about 9 MPa, which is adequate for working in DMFC. No significant increase in this efficiency factor was recorded with a further increase in amount of PAMPS. Accordingly, a molar ratio 1:1 of PVA and PAMPS is the best ratio to develop polyelectrolyte blend membranes as a possible substitute for Nafion in DMFCs applications.

## Figures and Tables

**Table 1:** Variation of residual weight percentage with temperature in TGA thermograms for non-cross-linked PVA and GA-cross-linked PVA, and PVA/PAMPS blend-based membranes.

Temp. (°C)	Residual Weight Percentage						
	Non-Cross-Linked PVA	GA-Cross-Linked PVA	GA-Cross-Linked Polymer Blends at Different PVA/PAMPS Ratio				
			1:0.5	1:1	1:1.5	1:2	1:3
100	90.15	92.51	85.72	83.33	77.24	74.36	70.02
200	85.17	90.91	81.86	80.33	75.54	70.66	68.70
300	55.90	60.12	57.24	50.16	46.61	44.02	40.73
400	38.14	34.54	30.86	29.37	30.15	22.42	21.95
500	25.80	20.11	25.5	24.51	22.08	24.46	23.91
600	10.51	12.96	10.34	8.20	7.99	7.76	5.15

**Table 2:** Thickness, tensile strength, elongation at break, contact angle, WU, MU, IEC, methanol permeability, and efficiency factor of the GA-cross-linked PVA and PVA/PAMPS blend-based membranes.

Membrane	Thickness (μm)	TS (MPa)	Elongation at Break (%)	Contact Angle	WU (%)	MU (%)	IEC (meq/gm)	Methanol Permeability Coefficient (cm^2^ s^−1^)	Efficiency Factor
GA-cross-linked PVA	181 ± 0.8	20.00 ± 1.6	53.97 ± 1.6	34.05 ± 1.6	60 ± 4	17.54 ± 1.6	0.077 ± 0.01	4.8 × 10^−7^	1.10 × 10^5^
PVA:PAMPS 1:0.5	184 ± 1.6	10.52 ± 0.81	109.08 ± 4	31.52 ± 2.4	65.26 ± 3.2	15.14 ± 2.4	1.23 ± 0.08	2 × 10^−8^	1.78 × 10^5^
PVA:PAMPS 1:1	187 ± 1.6	9.18 ± 0.81	126.34 ± 3.3	25.42 ± 1.6	70.51 ± 4	13.04 ± 1.6	2.64 ± 0.16	2.5 × 10^−8^	4.00 × 10^5^
PVA:PAMPS 1:1.5	188 ± 2.4	8.37 ± 0.41	162.18 ± 4	23.15 ± 0.8	73.36 ± 2.4	11.88 ± 2.4	3.587 ± 0.16	2.8 × 10^−8^	4.10 × 10^5^
PVA:PAMPS 1:2	190 ± 1.6	7.73 ± 0.41	32.27 ± 1.6	20.73 ± 0.8	75.86 ± 4	10.54 ± 3.3	3.77 ± 0.32	3.0 × 10^−8^	4.57 × 10^5^
PVA:PAMPS 1:3	192 ± 1.6	3.01 ± 0.33	14.56 ± 0.8	18.13 ± 1.60	90.16 ± 3.27	9.51 ± 4	3.93 ± 0.36	3.2 × 10^−8^	4.70 × 10^5^
Nafion-117	185	18.20	12.2	110	18.94	22	0.91	3.39 × 10^−6^	2.6 × 10^5^

**Table 3:** Cost of Nafion 117, pristine PVA, PAMPS, and PVA:PAMPS (1:1) blend-based membranes.

Sample (Sigma-Aldrich)	Nafion 117	PVA	PAMPS	PVA–PAMPS 1:1 (10 × 10 cm)
Cost	(10 × 10 cm)/$33.00	(25 G)/$47.07	(100 G)/$136.36	$3.2464
		(1 G)/$1.882	(1 G)/$1.3636	
